# An Oncology Urgent Care Clinic for the Management of Immune-Related Adverse Events: A Descriptive Analysis

**DOI:** 10.3390/curroncol29060347

**Published:** 2022-06-17

**Authors:** Kai-li Liang, Sean Tackett, Samantha Myers, Julie R. Brahmer, Ilene S. Browner, David S. Ettinger, Patrick M. Forde, Russell K. Hales, Christine L. Hann, Vincent K. Lam, Kristen A. Marrone, Tricia Patel, Valerie Peterson, Sarah Sagorsky, Michelle Turner, Khinh R. Voong, Jarushka Naidoo, Josephine L. Feliciano

**Affiliations:** 1 Sidney Kimmel Comprehensive Cancer Center, Johns Hopkins University, Baltimore, MD 21287, USA; kliang14@jhmi.edu (K.-l.L.); smyers46@jhmi.edu (S.M.); brahmju@jhmi.edu (J.R.B.); ibrowner@jhmi.edu (I.S.B.); ettinda@jhmi.edu (D.S.E.); pforde1@jhmi.edu (P.M.F.); rhales1@jhmi.edu (R.K.H.); chann1@jhmi.edu (C.L.H.); vklam@jhmi.edu (V.K.L.); kmarron1@jhmi.edu (K.A.M.); tbull2@jhmi.edu (T.P.); vrowe4@jhmi.edu (V.P.); sboneri1@jhmi.edu (S.S.); mturne42@jhmi.edu (M.T.); kvoong1@jhmi.edu (K.R.V.); jnaidoo1@jhmi.edu (J.N.); 2 Division of General Internal Medicine, Johns Hopkins Bayview Medical Center, Baltimore, MD 21224, USA; stacket1@jhmi.edu; 3 Biostatistics, Epidemiology and Data Management Core, Johns Hopkins School of Medicine, Baltimore, MD 21287, USA; 4 Bloomberg-Kimmel Institute for Cancer Immunotherapy, Johns Hopkins University, Baltimore, MD 21287, USA; 5 Department of Oncology, Beaumont Hospital Dublin, RCSI University of Health Sciences, D02 YN77 Dublin, Ireland

**Keywords:** immune-related adverse events, urgent care, care delivery, immune-checkpoint inhibitors, toxicity

## Abstract

Introduction: With the increasing use of immune checkpoint inhibitors (ICI) for cancer, there is a growing burden on the healthcare system to provide care for the toxicities associated with these agents. Herein, we aim to identify and describe the distribution of encounters seen in an urgent care setting for immune-related adverse events (irAEs) and the clinical outcomes from irAE management. Methods: Patient demographics, disease characteristics, and treatment data were collected retrospectively from encounters at an oncology Urgent Care Clinic (UCC) from a single tertiary center for upper aerodigestive malignancies from 1 July 2018 to 30 June 2019. Data were summarized using descriptive statistics with odds ratios for associations between patient features and hospitalization after UCC evaluation. Results: We identified 494 encounters from 289 individual patients over the study period. A history of ICI therapy was noted in 34% (n = 170/494) of encounters and 29 encounters (29/170, 17%) were confirmed and treated as irAEs. For those treated for irAEs, the majority (n = 19/29; 66%) were discharged home. Having an irAE was associated with an increased risk of hospitalization compared to non-irAEs (OR 5.66; 95% CI 2.15–14.89; *p* < 0.001). Conclusion: In this single institution experience, the majority of UCC encounters for confirmed irAEs were safely managed within the UCC. In ICI-treated patients, having an irAE was associated with an increased risk of hospitalization versus non-irAEs.

## 1. Introduction

**Figure 1:**
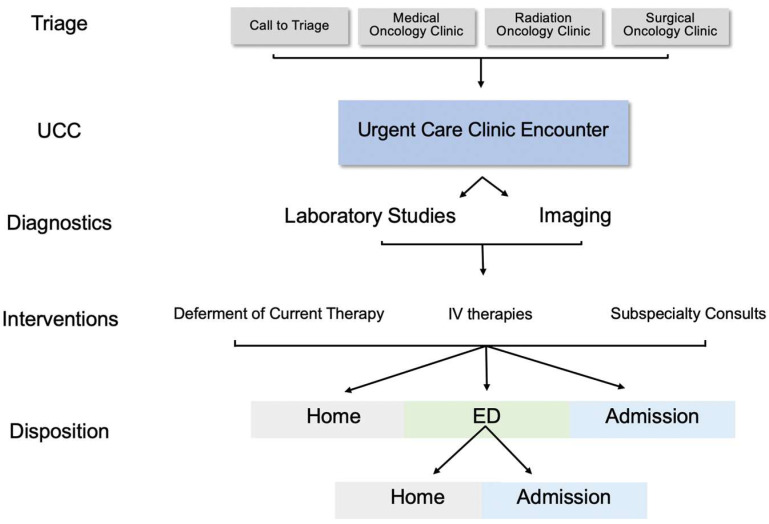
Urgent care clinic encounter work flow.

Over the past decade, several oncology centers created dedicated urgent care clinics (UCCs) as intermediaries between the clinic and the Emergency Department (ED) or inpatient care. Oncology UCCs demonstrate adequate triaging and potentially reduce overall ED visits without delays in care [1,2,3]. In 2018, the Sidney Kimmel Thoracic Oncology Clinic at Johns Hopkins Bayview Medical Center (JHBMC) created the UCC co-located in the cancer clinic. A telephone triage nurse refers patients from home or providers refer from one of the cancer center clinics which include medical, radiation, and surgical oncology. The UCC is staffed by one oncology nurse practitioner, and one nurse with the capacity for one urgent care slot each hour, Monday-Friday from 8 am to 3 pm; closing at 6 pm to allow for patient workup to be completed. The UCC providers can obtain urgent laboratory tests and imaging with equal priority status as those from the ED and can administer intravenous (IV) infusions. The UCC staff can also transfer patients via stretcher to the adjoining Johns Hopkins Bayview Hospital via direct admission or though the ED (Figure 1). Moreover, the UCC was established with the goal of expediting diagnostics and interventions, avoiding patient exposure to the ED, and reducing unnecessary hospitalizations. One perceived benefit of UCCs is the identification of treatment-related toxicities, including immune checkpoint inhibitors (ICI). Healthcare systems increasingly need to identify and manage immune-related adverse events (irAEs), which are unique and require different diagnostic and management pathways often involving non-oncology subspecialists and services [1,2,4]. In some reports, 13–32% of ED presentations are for confirmed irAEs in patients receiving ICIs [5,6]. Anti-PD-(L)1, and anti-CTLA-4 ICIs can result in a range of irAEs with the onset of irAEs ranging from days to years after initial therapy and can mimic other common conditions such as infectious, pulmonary, or cardiac conditions [5,7,8]. Thus, the diagnosis and management of complex irAEs often involve multi-disciplinary input from oncologists and subspecialist providers [9,10]. Little is known about the role of UCCs in diagnosis and management of irAEs and its association with subsequent hospital resource utilization or clinical outcomes [11]. In this study, we aim to give a descriptive analysis of patients with immune mediated toxicities in a dedicated oncology UCC. 

## 2. Materials and Methods

### 2.1. Patient Encounters and Demographics

We identified patient encounters at the Sidney Kimmel Thoracic Oncology Clinic at Johns Hopkins Bayview from 1 July 2018 to 30 June 2019, under an institutional review board approved protocol. We analyzed outcomes by encounter rather than by individual patient as we aimed to evaluate diagnoses, interventions, and dispositions (home vs. ED vs. direct admission) from each visit. Of note regarding the distinction between ED and direct admission, the UCC policy is such that peri-stable patients who may need Intermediate Level of Care (IMC) or Intensive Care Unit (ICU) are transferred to the ED for acute resuscitation or stabilization (e.g., use of vasopressors or mechanical ventilation). Direct admission to the floor is generally reserved for patients who have relatively stable vitals but cannot be discharged home. Direct admission vs. ED may also be limited by inpatient bed availability. We completed chart review and collected demographic, clinical, and disposition data for each UCC encounter through manual chart review. Data included age, sex (male, female), race subdivided into White/Caucasian, Black/African American, and Other (Hispanic, Asian/Pacific Islander, Native American/Alaskan), Eastern Cooperative Oncology Group Performance Scale (ECOG PS), Stage I–IV, Oncologic/Hematologic Primary (Lung, Esophageal, Other (see Table 1 footnote), and smoking status (never, former, current). Prior treatment (ICI monotherapy, ICI in Combination with Chemotherapy, ICI Clinical Trial, ICI Standard of Care (SOC), prior irAE. Urgent Care Disposition (Home, ED to Admit, ED to Home, Direct Admit) were also collected.

### 2.2. irAE Definition and Data Collection

We verified an irAE as related to ICI therapy by review of hospitalization course and outpatient follow-up documentation up to 1 month following the UCC encounter. We identified and confirmed irAEs by chart review of UCC provider notes by two independent chart reviewers (KL, JF) by evaluating clinical, radiographic, and pathologic irAE data when available, as well as irAE-directed therapies received (e.g., systemic steroids, IVIG). Severity of irAEs were evaluated by on Common Terminology Criteria for Adverse Events (CTCAE) Version 5.0, based on documented clinical notes or retrospective determination by chart reviewers (KL, JF) [12]. A sequalae or recurrence of a prior irAE was defined as an irAE that had been diagnosed prior to UCC encounter and had begun initial management, however, worsened leading to the documented UCC encounter.

### 2.3. Statistical Analysis

We summarized study data using descriptive statistics. Bivariate associations were calculated parametric and non-parametric tests as appropriate. T-tests were used for age and chi squared or Fisher’s exact test for categorical variables. Fisher’s exact test was used where a cell had less than ten counts. We performed a series of logistic regressions to derive odds ratios using hospitalization and irAEs as outcomes. The regressions used patient and treatment features. These variables were evaluated for associations with hospitalization after urgent care evaluation and were adjusted for clustering of characteristics within patients using multilevel mixed effects logistic regression models. Unknown or N/A responses were treated as missing in the statistical analyses. *p* values were reported as Wald statistics. Statistical analyses were performed using STATA 13 software (StataCorp. 2013. Stata Statistical Software: Release 13. College Station, TX, USA: StataCorp LP). 

## 3. Results

**Figure 2:**
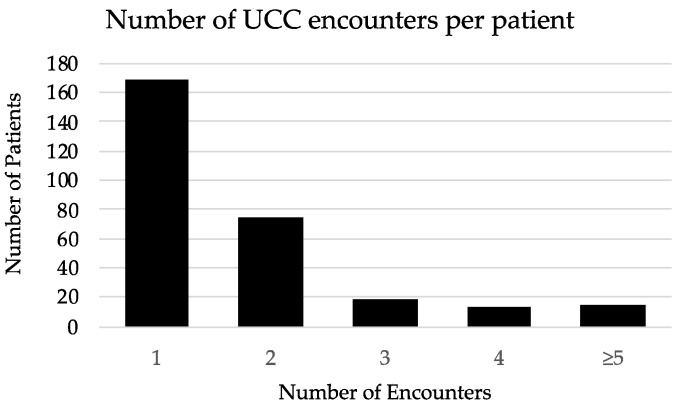
Histogram of urgent care clinic encounters per patient.

We identified 494 urgent care encounters occurring between 1 July 2018 and 30 June 2019 at the JHBMC Oncology UCC from 289 individual patients. The median number of encounters per patient was 1 encounter (interquartile range (IQR) 1 encounter) (Figure 2). Of these, 170 encounters (34%) were from 99 patients that had a history of receipt of ICI therapy (Table 1). Of these 170 patient encounters, 56% (n = 96) were actively receiving ICI at the time of UCC presentation, while 44% (n = 74) had either completed or stopped treatment. Those who had received ICI as combination chemotherapy-ICI accounted for 66% (n = 113) while ICI monotherapy accounted for 34% (n = 57). The median was four doses of ICI (IQR 5.5 doses) prior to UCC visit. A total of 35 encounters (21%) included patients who received ICI as part of a clinical trial.

### 3.1. UCC Encounter Demographics

Table 1 shows the demographic and clinical distribution of all UCC patient encounters. The majority of encounters represented patients of White/Caucasian race (n = 385; 78%), and patients most commonly had primary lung cancer, (n = 346; 70%) followed by esophageal cancer (n = 70; 14%), had Stage IV disease (n = 230; 47%), and an ECOG PS of 0–1 (n = 274; 55%). There was no difference in the mean age at UCC visit for encounters with ICI-treated patients versus non-ICI treated patients and no age difference between those with confirmed irAE versus those without irAE. 

### 3.2. Evaluation of Patients with a History of ICI

The most common presenting complaints for those who had ever received ICI were dyspnea (29%), fever (15%), pain (15%), GI symptoms (14%), dehydration/fatigue (11%), and asymptomatic laboratory abnormality (4%), or other causes (12%). Diagnostic studies included same day laboratory studies (n = 97; 57%) and imaging (n = 33; 54%). The most common imaging performed during the UCC visit included computed tomography (CT) (91%) to evaluate for radiographic evidence of an irAE. Out of the 33 encounters (58%) that had same day imaging, imaging was able to rule out irAEs in 19 encounters (58%). IrAEs were diagnosed clinically (52%), radiographically (38%), or from laboratory data (10%); 7% were eventually pathologically confirmed. Other reasons for UCC visits among ICI patients included complications of primary cancer (9%), systemic chemotherapy effects (18%), infection (19%), concurrent chemotherapy complication and primary cancer complications (28%), and non-oncologic presentations (8%). 

Therefore, of 170 encounters with ICI-treated patients, 29 (17%) had confirmed irAEs. The cumulative incidence among ICI-treated patients seen in the UCC was 17% over 1 year, or 5.87% of all UCC visits (Table 1). 

### 3.3. Distribution of irAEs 

Most confirmed irAEs were high grade (grade ≥ 3) (59%). The majority of encounters represented patients being treated with standard of care ICIs (72%), as opposed to those being treated on clinical trial (28%). The majority of irAEs were in patients who had received ICI in combination with chemotherapy (69%). Pneumonitis was the most common irAE (n = 11; 38%), followed by dermatitis (n = 9; 31%), hypophysitis (n = 3; 10%), colitis/diarrhea (n = 2; 7%), and hepatitis (n = 2; 7%), with singular cases of arthritis, and pancreatitis. Of the 29 encounters with confirmed irAEs, 11 (38%) encounters had a history of a prior irAE with a median of 56 days (IQR 67 days) since the prior irAE. The majority (n = 21; 72%) of irAEs were new, while 28% (n = 8) were for sequelae of or recurrence of prior irAEs. For newly diagnosed irAEs, the median number of ICI doses prior to irAE was 3.5 doses (IQR 6 doses) with a median of 68 days (IQR 168 days) since the initiation of ICI therapy to iAE encounter. For patients with a confirmed irAE, the most common ICIs were durvalumab (n = 8/29; 28%) and pembrolizumab (n = 10/29; 34%). In our observations for irAEs, 70% (n = 7) of those hospitalized and 32% (n = 6) of those managed as outpatient required subspecialty consults including pulmonology, endocrinology, gastroenterology, rheumatology, cardiology, dermatology, and neurology.

### 3.4. Management, and Outcomes for irAEs by Disposition

For those encounters diagnosed with confirmed irAEs, 66% (n = 19) were discharged home, 14% (n = 4) were directly admitted and 21% (n = 6) were referred to the ED and were subsequently admitted, totaling 10 (34%) hospitalizations (Table 1).

#### 3.4.1. Outpatient Management

For those irAE encounters that resulted in home discharges, the toxicities were mainly low-grade: grade 1 [dermatitis (n = 2)], grade 2 [dermatitis (n = 5), pneumonitis (n = 4), arthritis (n = 1)], and grade 3 [dermatitis (n = 2), pneumonitis (n = 2), colitis (n = 1), hypophysitis (n = 1), hepatitis (n = 1)]. 

Interventions in the UCC included the administration of IV fluids (11%) and intravenous immunoglobulin (IVIG) (5%) for irAEs. None received IV antimicrobials or IV steroids; however, most were prescribed oral steroids (58%). A select group of patient encounters (31%) had their current therapy held following evaluation in the UCC. The majority (n = 15; 79%) of irAEs discharged home improved or resolved and followed up in clinic within a median of 3 days (IQR 5 days). For those cases that did not improve or worsened following discharge to home (n = 4/19), all were grade 3 irAEs and all resulted in hospitalization within 7 days of the UCC encounter. 

#### 3.4.2. Inpatient Management

For those encounters resulting in hospitalization via direct admission or via the ED, the toxicities at UCC presentation were as follows: grade 3 [pneumonitis (n = 5), hypophysitis (n = 2), hepatitis (n = 1), pancreatitis (n = 1), colitis/diarrhea (n = 1)]. Two of the hospital admissions were for sequelae/recurrence of prior irAEs; however, the majority (n = 8; 80%) were for new irAEs. All irAEs requiring hospitalization received corticosteroids during their admission and two encounters with pneumonitis received IVIG after corticosteroids. All ten encounters improved and were discharged. Following discharge, of the eight encounters who had patients receiving ICI at the time of irAE diagnosis, all discontinued their ICI therapy indefinitely. 

### 3.5. Associations between Encounter Features and UCC Visit Disposition

**Figure 3:**
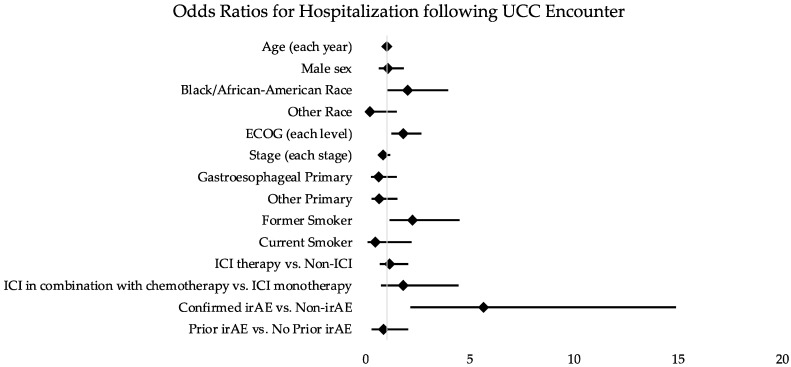
Odds ratios for clinical features and hospitalization following UCC encounters. Abbreviations: OR (odds ratio); ED (emergency department); UCC (urgent care clinic); ICI (immune-checkpoint inhibitor); irAE (immune-related adverse event); ECOG (Eastern Cooperative Oncology Group).

**Figure 4:**
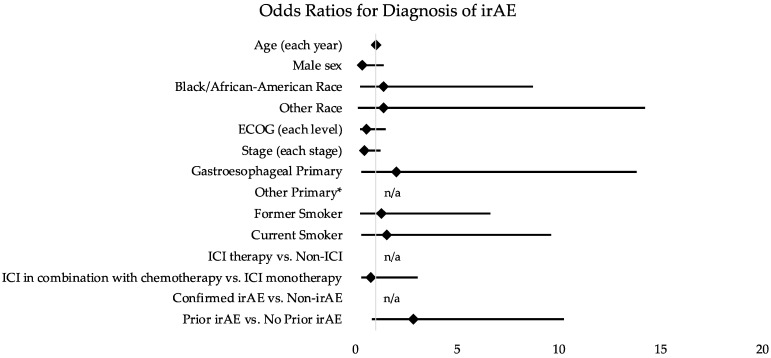
Odds ratios for clinical features and diagnosis of irAE. Abbreviations: OR (odds ratio); ED (emergency department); UCC (urgent care clinic); ICI (immune-checkpoint inhibitor); irAE (immune-related adverse event); ECOG (Eastern Cooperative Oncology Group). * Could not be calculated because there were no observed irAEs for “Other Primary”.

Having an irAE was associated with an increased risk of hospitalization compared to non-irAEs (OR 5.66; 95% CI 2.15–14.89; *p* < 0.001). ICI therapy itself was not associated with increased risk for hospitalization (OR 1.15; 95% CI 0.66–2.03; *p* = 0.620) (Table 2). There was an association between increasingly poor ECOG PS (OR 1.81; 95% CI 1.23–2.67; *p* = 0.003), former smoking history (OR 2.25; 95% CI 1.13–4.49; *p* = 0.021), and Black/African American race (OR 2.00; 95% CI 1.01–3.96; *p* = 0.048) with risk of hospitalization (Table 2; Figure 3). We did not identify any clinical or demographic features that were associated with an increased risk of irAE diagnosis (Table 3; Figure 4). 

## 4. Discussion

### 4.1. Main Findings

To our knowledge, this is the first study aimed at describing the role of an oncology UCC in the diagnosis and management of irAEs. 

Over one-third of all UCC encounters included patients who had ever received ICIs and 17% of UCC encounters in this cohort represented confirmed irAEs. While we observed that a confirmed irAE was associated with an increased risk for hospitalization, reassuringly the majority of UCC visits for patients with irAEs were able to be managed in the UCC setting and were subsequently discharged home from UCC regardless of grade. This observation may be attributed to the variety of services offered by the UCC, including expedited diagnostics and therapies such as IV fluids, IVIG infusions, services that are difficult to coordinate in a traditional outpatient setting; however also do not require hospitalization. 

### 4.2. Comparison with Other Studies

We reported that 17% of our UCC encounters resulted in irAE diagnosis. Similarly, several studies have reported that 13 to 32% of ED visits for patients on ICI therapy are for irAEs [5,6,13]. This suggests that the role of the UCC may be to help decompress some of the clinical volume in the ED and that only the most clinically appropriate needing higher levels of care are sent through the ED for admission. 

We reported a higher incidence of pneumonitis (38%) in our UCC compared to prior studies evaluating irAEs through the ED (5–26%) and we observed that only 7% of our cohort presented to UCC with colitis, while published data suggests this may be as high as 12–14% of hospitalizations [11,13,14]. This is explained by the location of our UCC, which is located in a primarily thoracic oncology clinic, thus these results may not apply to other cancers treated with ICI. 

In the prior literature, the percentage of hospitalizations for irAE that prompted subspecialty consults was 91% [11]. In our observations for irAEs, 70% of those hospitalized and 32% of those managed as outpatient required subspecialty consults. These data further emphasize the role and importance of multidisciplinary immunotherapy toxicity teams and cross-specialty training in the identification and management of irAE [10,15]. We also reported an association with demographic and clinical factors associated with hospitalization after urgent care including smoking status, race, and performance status. These factors may be important to study further to identify other patient factors associated with higher risks of complications or inferior clinical outcomes. 

### 4.3. Limitations

Our study has several limitations. First, this is a retrospective study and descriptive analysis without a comparison group and so our ability to compare clinical outcomes between UCC and ED are limited. Furthermore, this study evaluated each UCC encounter, rather than each patient, and several patients had multiple encounters including some for the same irAE and others for different complaints. The 29 confirmed encounters for irAEs were from 24 individual patients. Four patients had two encounters for recurrence of the same irAE and one patient had encounters for two separate irAEs. This potentially overestimates the incidence of irAEs; however, it also demonstrates irAE recurrence and multisystem irAEs, which are recognized phenomena [16]. 

Additionally, while irAE grading is ideally completed prospectively, we attempted to account for our retrospective assessment by having two independent clinicians grade irAE events. Protocols to improve documentation and grading of possible irAEs during the UCC visit, may also serve to alert providers as to when hospitalization is necessary. This real-time documentation can also potentially improve communication with ED or inpatient teams, minimizing diagnostic delays and leading to earlier activation of subspecialty consults or toxicity teams [15].

### 4.4. Implications and Future Directions

Despite these limitations, our study’s findings may have future implications for practices and procedures both within our institution and beyond. For example, we observed that grade ≥ 3 irAEs, often required further evaluation in an ED setting or with inpatient admission; however, for grade 1–2 irAEs, outpatient management with oral steroids or subspecialty consults may be appropriate. Drawing from the observations of the four encounters with grade 3 events that resulted in home discharges, but subsequently worsened, policies could be created that require certain grade 3 irAEs, such as pneumonitis, regardless of clinical stability, to be admitted from UCC rather than be managed as an outpatient. From our observations, irAE-specific best-practice guidelines may be designed for future outpatient management.

Our institution recently implemented a system-wide automated protocol to flag patient charts indicating a history of receiving immunotherapy and simultaneously prompts consideration for irAEs. We have demonstrated that many irAEs also require subsequent subspecialty consultation. Alerts and direct lines of communication with specialized toxicity teams could assist in early diagnosis and management of irAEs. Additional future directions include creating algorithms to aid in triage, diagnosis, and management for patients on ICI to help determine appropriate disposition. The proposed benefits of these algorithms would be to direct patients to a dedicated oncology UCC and off-load crowded EDs thus avoiding exposure of immunocompromised patients to the general hospital populations. In addition to these algorithms, evidence-based order sets can be designed for diagnostic evaluation and management of irAEs. These order sets, including various labs and imaging orders, could be piloted in the UCC, and then subsequently applied to ED and inpatient settings. 

Further studies will be essential to determine how irAE clinics will impact on the diagnosis and workflow management of irAEs and influence outcomes. 

## 5. Conclusions

In this single institution experience, the majority of UCC encounters for confirmed irAEs were safely managed within the UCC. For encounters with ICI-treated patients, having an irAE was associated with an increased risk for requiring hospitalization compared to non-irAEs. Ultimately, further studies will be needed to assess the role of UCCs in the diagnosis and management of irAEs. 

## Figures and Tables

**Table 1:** Demographic data for urgent care clinic patient encounters from 2018–2019.

Demographic Characteristic	Non-ICI Therapy n = 324 (%)	ICI Therapy n = 170 (%)	*p*	ICI Therapy		*p*
				Non-irAE n = 141 (%)	irAE n = 29 (%)	
Age, years, mean ± SD	63.8 ± 10.8	65.5 ± 9.5	0.082	65.3 ± 9.6	66.9 ± 9.0	0.408
Sex						
Female	169 (52)	79 (46)	0.230	62 (44)	17 (59)	0.150
Male	155 (48)	91 (54)		79 (56)	12 (41)	
Race						
White/Caucasian	258 (80)	127 (75)	0.121	107 (76)	20 (69)	0.617
Black/African-American	48 (15)	25 (15)		20 (14)	5 (17)	
Other	18 (5)	18 (11)		14 (10)	4 (14)	
ECOG score (prior to UCC encounter)						
0–1	157 (48)	117 (69)	0.071	96 (68)	21 (72)	0.926
2	70 (22)	47 (28)		40 (28)	7 (24)	
3+	23 (7)	6 (4)		5 (4)	1 (3)	
Stage (at UCC encounter)						
I	13 (4)	1 (0)	0.009	1 (0)	0 (0)	0.152
II	24 (7)	14 (8)		9 (6)	5 (17)	
III	107 (33)	50 (29)		41 (29)	9 (31)	
IV	129 (40)	101 (59)		88 (62)	13 (45)	
Oncologic/Hematologic Primary						
Lung	198 (61)	148 (87)	<0.001	125 (89)	23 (79)	0.345
Esophageal	49 (15)	21 (12)		15 (10)	6 (21)	
Other *	77 (24)	1 (0)		1 (0)	0 (0)	
Smoking Status						
Never	116 (36)	29 (17)	<0.001	25 (18)	4 (14)	0.366
Former	171 (53)	124 (73)		104 (74)	20 (69)	
Current	37 (11)	17 (10)		12 (9)	5 (17)	
Prior Therapy						
ICI Monotherapy	n/a	57 (34)	n/a	48 (34)	9 (31)	0.832
ICI in Combination with Chemotherapy	n/a	113 (66)		93 (66)	20 (69)	
ICI Clinical Trial	0 (0)	35 (21)	n/a	27 (19)	8 (28)	0.318
ICI SOC	0 (0)	135 (79)		114 (81)	21 (72)	
Prior irAE	0 (0)	37 (22)	<0.001	26 (18)	11 (38)	0.021
Urgent Care Disposition						
Home	279 (86)	147 (86)	0.226	128 (91)	19 (66)	0.002
ED to Admit	27 (8)	12 (7)		6 (4)	6 (21)	
ED to Home	8 (2)	1 (0)		1 (0)	0 (0)	
Direct Admit	10 (3)	10 (6)		6 (4)	4 (14)	

Abbreviations: UCC (urgent care clinic); ICI (immune-checkpoint inhibitor); irAE (immune-related adverse event); ECOG (Eastern Cooperative Oncology Group); SOC (standard of care); n/a (non-applicable). * Other oncologic and hematologic primary conditions included colon cancer, gallbladder cancer, pancreatic cancer, cholangiocarcinoma, breast cancer, thymoma, follicular lymphoma, diffuse large b-cell lymphoma, CLL, multiple myeloma, renal cell carcinoma, iron deficiency anemia, ITP, recurrent DVT/PE.

**Table 2:** Associations between clinical features and hospitalization following UCC encounters.

Clinical Demographic	OR: Outcome Hospitalization (ED to Admit and Direct Admit)	95% CI
Age (each year)	1.02	0.99–1.05
Sex		
Female (ref)	-	
Male	1.05	0.61–1.82
Race		
White/Caucasian (ref)	-	
Black/African American	2.00	1.01–3.96
Other	0.20	0.03–1.47
ECOG (prior to UCC encounter) (each level)	1.81	1.23–2.67
Stage (at UCC visit) (each stage)	0.82	0.58–1.17
Oncologic/Hematologic Primary		
Lung (ref)	-	
Esophageal ^1^	0.61	0.25–1.49
Other ^2^	0.64	0.27–1.52
Smoking Status		
Never Smoker (ref)	-	
Former Smoker ^3^	2.25	1.13–4.49
Current Smoker ^4^	0.47	0.10–2.19
ICI therapy vs. Non -ICI	1.15	0.66–2.03
ICI in combination with chemotherapy vs. ICI monotherapy	1.79	0.72–4.44
Confirmed irAE vs. Non-irAE	5.66	2.15–14.89
Prior irAE vs. No Prior irAE	0.88	0.28–2.71

^1^ “Other” treated as missing; ^2^ “Esophageal” treated as missing, ^3^ “Current” treated as missing, ^4^ “Former” treated as missing. Abbreviations: OR (odds ratio); ED (emergency department); UCC (urgent care clinic); ICI (immune-checkpoint inhibitor); irAE (immune-related adverse event); ECOG (Eastern Cooperative Oncology Group); “-” indicates no data provided given it was the reference value.

**Table 3:** Associations between clinical features and diagnosis of irAE.

Clinical Demographic	OR: Outcome Confirmed irAE	95% CI
Age (each year)	1.02	0.95–1.09
Sex		
Female (ref)	-	
Male	0.31	0.07–1.39
Race		
White (ref)	-	
Black/African American	1.39	0.22–8.72
Other	1.37	0.13–14.25
ECOG (prior to UCC encounter) (each level)	0.55	0.20–1.48
Stage (at UCC visit) (each stage)	0.44	0.16–1.21
Oncologic/Hematologic Primary		
Lung (ref)	-	
Esophageal ^1^	2.01	0.29–13.79
Other ^2^	n/a	n/a
Smoking Status		
Never Smoker (ref)	-	
Former Smoker ^3^	1.27	0.24–6.62
Current Smoker ^4^	1.56	0.25–9.60
ICI therapy vs. Non-ICI	n/a	n/a
ICI in combination with chemotherapy vs. ICI monotherapy	0.73	0.27–3.08
Confirmed irAE vs. Non-irAE	n/a	n/a
Prior irAE vs. No Prior irAE	2.87	0.80–10.26

^1^ “Other” treated as missing; ^2^ “Esophageal” treated as missing, ^3^ “Current” treated as missing, ^4^ “Former” treated as missing. Abbreviations: OR (odds ratio); ED (emergency department); UCC (urgent care clinic); ICI (immune-checkpoint inhibitor); irAE (immune-related adverse event); ECOG (Eastern Cooperative Oncology Group); n/a (non-applicable), “-” indicates no data provided given it was the reference value.

## Data Availability

The data presented in this study are available on request from the corresponding author. The data are not publicly available to ensure and maintain the privacy and confidentiality of individuals’ health information.
